# A Review of the Use of Extracellular Vesicles in the Treatment of Neonatal Diseases: Current State and Problems with Translation to the Clinic

**DOI:** 10.3390/ijms25052879

**Published:** 2024-03-01

**Authors:** Kirill Goryunov, Mikhail Ivanov, Andrey Kulikov, Yulia Shevtsova, Artem Burov, Yulia Podurovskaya, Victor Zubkov, Dmitry Degtyarev, Gennady Sukhikh, Denis Silachev

**Affiliations:** 1 V.I. Kulakov National Medical Research Center for Obstetrics, Gynecology and Perinatology, Moscow 117198, Russia; k_gorunov@oparina4.ru (K.G.); neokarda@mail.ru (M.I.); yulshevtsova@yandex.ru (Y.S.); burovmd@gmail.com (A.B.); podurovskaya@yandex.ru (Y.P.); viktor.zubkov@mail.ru (V.Z.); d_degtiarev@oparina4.ru (D.D.); g_sukhikh@oparina4.ru (G.S.); 2 A.N. Belozersky Institute of Physico-Chemical Biology, Lomonosov Moscow State University, Moscow 119992, Russia; 3 Medical Institute, Patrice Lumumba Peoples’ Friendship University of Russia (RUDN University), Moscow 117198, Russia; kulikov-anv@rudn.ru

**Keywords:** extracellular vesicles, mesenchymal stromal cells, neonatal pathologies, premature newborns, cell therapy

## Abstract

Neonatal disorders, particularly those resulting from prematurity, pose a major challenge in health care and have a significant impact on infant mortality and long-term child health. The limitations of current therapeutic strategies emphasize the need for innovative treatments. New cell-free technologies utilizing extracellular vesicles (EVs) offer a compelling opportunity for neonatal therapy by harnessing the inherent regenerative capabilities of EVs. These nanoscale particles, secreted by a variety of organisms including animals, bacteria, fungi and plants, contain a repertoire of bioactive molecules with therapeutic potential. This review aims to provide a comprehensive assessment of the therapeutic effects of EVs and mechanistic insights into EVs from stem cells, biological fluids and non-animal sources, with a focus on common neonatal conditions such as hypoxic–ischemic encephalopathy, respiratory distress syndrome, bronchopulmonary dysplasia and necrotizing enterocolitis. This review summarizes evidence for the therapeutic potential of EVs, analyzes evidence of their mechanisms of action and discusses the challenges associated with the implementation of EV-based therapies in neonatal clinical practice.

## 1. Introduction

The development of pathologies in newborns and premature infants is often attributed to prematurity and the complications associated with it. Prematurity often leads to early neonatal complications and increases the risk of adverse outcomes through the occurrence of severe pathologies. The World Health Organization reports that the incidence of preterm birth varies internationally, ranging from 4% to 15% of all births [1,2]. Neonatal pathologies include various conditions that affect infants within the first 28 days of life and can lead to long-term impairment or severe disability, depending on their severity. These diseases usually affect several organ systems and are often accompanied by extensive inflammatory processes. The most common neonatal pathologies include hypoxic–ischemic encephalopathy (HIE), respiratory distress syndrome (RDS), bronchopulmonary dysplasia (BPD), sepsis and necrotizing enterocolitis (NEC) [3].

Despite advances that have increased survival rates, complications such as chronic lung disease, brain injury and neonatal pulmonary hypertension remain common and pose a significant risk of permanent complications affecting the nervous system, lungs, heart and metabolism [4]. The treatment of neonatal pathologies requires an interdisciplinary approach involving a team of anesthesiologists, neonatologists, pediatricians, pediatric surgeons, neuropathologists, rehabilitation specialists and others to make early diagnoses and implement innovative treatments that complement traditional therapies [5,6]. Currently, pharmacologic interventions in the early perinatal period are some of the main therapeutic strategies, but they can have side effects and are not consistently effective. The pharmacological treatment of newborns poses a particular challenge as there are few drugs available that are tailored to this specific population group. Currently, there is a lack of drugs specifically formulated and researched for neonates, resulting in the frequent use of adult drugs without thorough investigations of their efficacy and safety in neonatal physiology. This practice can lead to the inappropriate administration of medication, incorrect dosing and inadequate timing, which can result in severe consequences and long-term disability. In addition, the immaturity of premature infants makes it difficult to assess the efficacy of medications as their pharmacokinetics and pharmacodynamics have not been extensively studied. Despite the urgent need for comprehensive research in this area, few studies have investigated the potential adverse effects of drugs in neonates. This underlines the urgent need for the appropriate categorization and validation of neonatal drugs, especially antibiotics, to prevent drug-related complications. [7]. Therefore, exploring novel treatments for acute neonatal diseases is critical for improving patient outcomes and reducing the incidence of neonatal disability. Preclinical and clinical studies of stem cell therapy have yielded encouraging results in a number of neonatal conditions, including chronic lung disease, intraventricular hemorrhage and HIE. These studies focus on the use of cells that have cytoprotective, reparative and regenerative properties [4,8,9]. The therapeutic effect of stem cells is thought to be primarily due to paracrine effects, largely mediated by the secretion of extracellular vesicles (EVs) [10]. EVs are considered a novel but under-researched therapeutic approach with similar efficacy to cell therapy. Current research on the biology of EVs suggests that these particles can regulate intercellular communication and transfer biologically active molecules to recipient cells. Although initial research focused on EVs from human and animal sources, including stem, progenitor and stromal cells, more recent findings have identified alternative EV sources such as plants, fungi and bacteria that offer additional opportunities for therapeutic applications [11,12].

The aim of this article is to review and critically analyze evidence of the biological effects and molecular mechanisms of EVs from various sources. It systematically summarizes evidence for the therapeutic potential of EVs, focusing on findings from animal studies in which neonatal pathology was modeled. It also discusses the challenges associated with the clinical translation of EV-based therapies in neonatology.

### 1.1. Searching Criteria

While conducting this review, we systematically searched scientific databases, including PubMed Central and the ClinicalTrials.gov database, from 1 January 2000 to 1 June 2023 (Figure S1). The first articles on the biogenesis mechanism of extracellular vesicles appeared in the year 2000. We therefore chose the year 2000 as the cut-off date. The following keywords were used in the search: ‘hypoxic-ischemic encephalopathy’, ‘respiratory distress syndrome’, ‘bronchopulmonary dysplasia’, ‘sepsis’, ‘necrotizing enterocolitis’, ‘cell therapy’, ‘extracellular vesicles’, ‘exosomes’, ‘microvesicles’, and ‘neonatology’. In this review, we refer to ‘extracellular vesicles’ in a broad sense. However, when specific terms such as ‘exosomes’ are mentioned, these are cases in which the authors provided convincing evidence that the particles have all the characteristics defined in the 2018 MISEV guidelines [13].

### 1.2. Applying the ‘Therapeutic Unit’ Concept to Assess the Equivalence of EVs Concentrations

Our analysis of the current literature revealed a variety of regimens and dosages for treatments using EVs, which makes the direct comparison of studies difficult. To address this issue, we used the concept of the ‘therapeutic unit’ proposed by Kordelas et al. [14] as a standard measure to normalize the administered doses of EVs. According to their work, one therapeutic unit corresponds to the amount of EVs produced by 4 × 10^7^ human bone marrow-derived MSCs within 48 h. This amount is approximately 1.3–3.5 × 10^10^ particles/unit and contains 0.5–1.6 mg protein/unit. Despite possible variations in cultivation conditions, we chose to convert all reported doses into therapeutic units using simple mathematical proportions. Furthermore, we assumed that EVs production under standard culture conditions (culture medium, temperature and CO_2_ concentration) was linear during the first 2–3 days, an assumption confirmed by unpublished data from our laboratory. However, this assumption should be taken with caution as the productive abilities of cells in vitro do not necessarily follow linear kinetics, and differences in laboratory protocols could influence EVs production rates.

By applying this standardization to the reported data, we aimed to make a more accurate comparison between studies and provide a speculative assessment of therapeutic effects depending on the EVs dose administered. However, it should be noted that our approach relies on several assumptions and simplifications that may affect the reliability of the resulting comparisons. Further standardization of methods for the preparation and quantification of EVs in this area is therefore essential to enable more robust analyses and comparisons between different studies.

## 2. Biogenesis and Classification of EVs

EVs are small particles consisting of a lipid bilayer that are released by cells into the extracellular space. EVs carry a cargo of proteins, nucleic acids and other biomolecules and play a crucial role in intercellular communication and the modulation of immune responses. The first mention of EVs was from Wolf P. in 1967, who obtained a pellet of EVs via the ultracentrifugation of platelet-poor plasma [15]. Later, it was shown that all cells release different types of EVs into the extracellular environment and participate in intercellular communication [16]. The initial classification of EVs was based on mechanisms of intracellular secretion and was categorized into three groups: (1) exosomes (diameter: 40–150 nm), which are secreted after the fusion of multivesicular bodies with the cell membrane; (2) microvesicles or ectosomes (diameter: 100–1000 nm), which are formed by budding from the plasma membrane into the intercellular space, and (3) apoptotic bodies (100–5000 nm), which are formed during apoptosis [17].

It should be noted that modern methods do not always allow for the isolation of pure EVs fractions and thus the determination of the intracellular origin of the analyzed vesicles [18]. This fact and the discovery of new types of EVs have led to the need to reconsider the classification of EVs. The International Society for Extracellular Vesicles has published recommendations called ‘minimal information for studies of EVs’ (MISEV) [13]. The recommendations suggest classifying EVs based on their physical characteristics: size, density, the presence of a lipid bilayer, biochemical properties (CD63+/CD81+, carrier of annexin A5, etc.) and according to their source and conditions of origin (EVs from podocytes, hypoxic EVs, large oncosomes, etc.). Therefore, the proposed nomenclature classifies small vesicles (20–200 nm), including exomers (≤50 nm) and supermers (≥25 nm) without a lipid bilayer, exosomes (40–130 nm) and defensosomes (approximately 80 nm). Large EVs with a diameter >200 nm include microvesicles (100–1 µm), migrasomes (500–3000 nm), apoptotic bodies (50 nm–5 µm) and large oncosomes (1–10 µm) [13,16].

Recent research has described a new type of small extracellular vesicles, in particular exomers and supermers, which are currently the least researched. Initial studies have shown that these small EVs contain a variety of cargoes, including proteins, nucleic acids and lipids. In addition, studies focusing on exomers have indicated an enrichment of proteins related to hypoxia, coagulation and metabolic pathways like glycolysis and the mTOR signaling pathway. On the other hand, supermers were found to exhibit significant enrichment of clinically relevant proteins such as amyloid precursor protein (APP), cellular mesenchymal epithelial transition factor (MET) and glypican 1 (GPC1). Overall, these results suggest that exomers and supermers are involved in intercellular communication and could potentially influence physiological processes within cells [19,20].

The most studied type of EVs are exosomes, whose biochemical properties ensure a high degree of cargo stability during transportation. The exosomal membrane is organized in the form of a lipid bilayer. It contains phospholipids, cholesterol and phosphatidylethanolamines which increase the stability of the exosome against the biodegradation of the cargo, thus providing efficient transport [21,22].

## 3. The Cargoes of EVs

**Figure 1:**
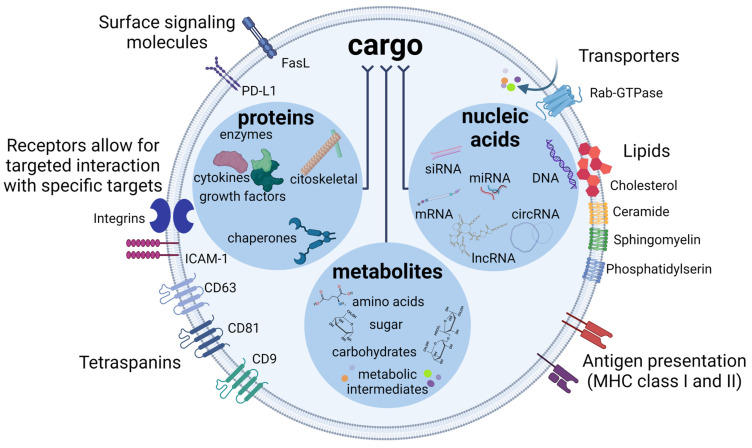
Detailed diagram of the architecture and composition of exosomes, highlighting the general structure and typical cargo components. Created using BioRender.com.

EVs are known to transport a variety of cargoes, including proteins, nucleic acids (various types of RNA and DNA), various carbohydrates and other biologically active molecules such as lipids (Figure 1). In some cases, organelles such as mitochondria or their components can also be detected in EVs [23,24]. Proteins and microRNAs (miRNAs) are thought to contribute significantly to the biological functions modulated by EVs. Current proteome and miRNA profiles are continuously integrated into online repositories such as EVpedia [25], ExoCarta [26] and Vesiclepedia [27], which facilitate subsequent bioinformatic analyses of the potential role of these macromolecules. In total, more than 1900 proteins have been identified in the proteome of bone marrow-derived MSC-EVs by mass spectrometry [28]. These proteins are functionally associated with self-renewal, differentiation, cell migration and proliferation. It has been observed that the proteome of MSC-EVs is enriched with proteins involved in angiogenesis under hypoxic conditions [29]. Moreover, a proteomic analysis of MSC-EVs from a human placenta revealed 745 proteins associated with functions related to angiogenesis, neurogenesis, immunoregulation, protection against apoptosis and oxidative stress. In particular, this analysis clearly identified proteins involved in the Wnt and phosphatidylinositol 3-kinase/protein kinase B (PI3K/Akt) signaling pathways, which have been shown to be associated with neuroprotection [30]. The neuroprotective properties appear to be related to the fundamental functions of MSCs. The proteome of MSC-EVs has been shown to confer neuroprotection through two primary mechanisms: first by promoting neuronal survival and enhancing neuroplasticity and second by mitigating inflammatory responses. MSC-EVs promote neuronal survival and neuroplasticity by transferring growth factors such as nerve growth factor (NGF), brain-derived neurotrophic factor (BDNF) and glial cell line-derived neurotrophic factor (GDNF) [31,32]. In addition, MSC-EVs have been found to modulate inflammation by regulating the activation state of immune cells through the secretion of anti-inflammatory cytokines such as interleukin 10 (IL-10), tumor growth factor β (TGF-β) and tumor necrosis factor-inducible gene 6 protein (TSG-6) [33]. Moreover, MSC-EVs are able to transfer interleukin-1 receptor antagonist protein (IL1-RA), which is crucial for blocking inflammation-induced injuries [34]. Most of all, during the incubation of bone marrow-derived MSCs with IFN-γ, these cells were shown to produce EVs containing a large amount of neuroprotective molecules (such as laminin β2, aggrecan, testican-1 and periostin) and a variety of miRNAs with anti-inflammatory effects, for instance, miRNA-200b and miRNA-146b [35]. Therapeutic effects have been shown to depend on the presence of both proteins and miRNAs in the contents of MSC-EVs. The degradation of miRNAs led to a reduction in observed neuroprotective effects in a murine model of autoimmune encephalomyelitis [35]. Moreover, a range of miRNAs associated with neuroprotection, such as miRNA-133b, miRNA-21-5p, miRNA-22-3p, miRNA-31 and miRNA-146a-5p, have been identified in the contents of human MSC-EVs [36,37,38,39,40].

It is known that EVs produced by MSCs and other cell types contain a diverse spectrum of RNA molecules. These include messenger RNA (mRNA), microRNA (miRNA), long non-coding RNA (lncRNA), small interfering RNA (siRNA), PIWI-interacting RNA (piRNA), ribosomal RNA (rRNA) fragments, Y-RNA, small nuclear RNA (snRNA) and circular RNA (circRNA) [41,42]. CircRNAs, a subgroup of non-coding RNAs, are generated via the non-sequential backsplicing of exons, introns or a combination of both. Their presence may facilitate the translation of unique small peptides through internal ribosome entry site-dependent mechanisms [43]. The regulation of the expression of this type of RNA has been shown to be crucial for the maintenance of normal neurogenesis [44]. CircRNAs have been shown to be involved in the regulation of autophagy, proliferation, apoptosis and the cell cycle [45]. At present, a link between the dysregulation of their expression and the progression of neurodegenerative [46] and cardiovascular [45] diseases has been established.

lncRNAs are RNA transcripts longer than 200 nucleotides and do not contain coding sequences for proteins. They are categorized into different groups, including sense, antisense, bidirectional, intronic and intergenic, based on their proximity to protein-coding transcripts. They are expressed at low levels and are unique for each cell type. It has been shown that lncRNAs play a certain role in regulating gene expression during development and differentiation at the post-transcriptional level by influencing chromatin structure and methylation, although the exact mechanisms are still unknown [47]. To date, only a small number of functional lncRNAs have been characterized, some of which have been found in human breast milk EVs. For example, certain lncRNAs such as growth arrest specific 5 (GAS5), steroid receptor activator 1 RNA (SRA1) and colorectal neoplasia differentially expressed (CRNDE) have been identified [47]. These lncRNAs play an important role in the regulation of neonatal metabolism. For example, GAS5 has been shown to inhibit the activation of glucocorticoid-responsive genes under conditions of starvation and cellular stress, which may be an adaptive mechanism to conserve energy resources in neonatal cells [48]. SRA1 acts as a coactivator of the transcriptional regulatory factor peroxisome proliferator-activated receptor gamma (PPARγ), which is responsible for adipogenesis [49]. CRNDE is involved in the regulation of signaling pathways triggered by insulin and insulin-like growth factor [50]. In addition, breast milk contains lncRNAs that are probably involved in the regulation of the neonatal immune system. For example, the lncRNA differentiation antagonizing nonprotein coding RNA (DANCR) has been shown to regulate the expression of *IL6* and tumor necrosis factor (*TNF*) genes in peripheral blood monocytes [51]. In addition, studies have shown that the lncRNA metastasis associated lung adenocarcinoma transcript (MALAT1), which is present in MSC-EVs, regulates various therapeutic targets, particularly inflammation, suggesting significant potential for the treatment of acute brain injury [52].

piRNAs are short sequences of 21 to 35 nucleotides that are associated with regulatory proteins from the PIWI family. These piRNA-PIWI complexes play a central role in maintaining the functional integrity of stem cells, mainly by suppressing retrotransposon mobilization in genomic sequences [53]. In particular, piRNAs such as hsa_piR_017723_DQ594464 and hsa_piR_020814_DQ598650 have been identified in MSC-EVs from human bone marrow. These piRNAs contribute to the protection of umbilical-cord blood derived stem cells from apoptosis [54].

Y-RNAs represent a relatively lesser-known class of non-coding RNAs. They have been observed in various human cell types, with particularly high levels of expression in brain and heart tissues [55]. To date, they have been found to regulate DNA replication, stabilize RNA transcripts and mediate defense mechanisms in response to cell stress [56]. For example, Y-RNA-1, which is highly enriched in MSC-EVs compared to the cells of origin, was found to play a crucial role in the protective effects of MSC-EVs on the TNF-α/ActD-mediated apoptosis of hepatocytes as the siRNA-mediated silencing of Y-RNA-1 reduces these effects in vitro [57]. It has also been shown that exosomes derived from cardiosphere-derived cells are enriched with small RNA components, predominant the Y RNA fragment EV-YF1, and provide therapeutic effects after myocardial infarction [58].

Current research clearly demonstrates the crucial function of lipids in the formation of the membrane architecture and their importance as biological signaling molecules. For example, cholesterol, which is transported in significant excess by EVs T-lymphocytes when transferred to peripheral blood mononuclear cells, contributes to their production of proinflammatory TNF-α [59]. EVs contain various lipid components such as sphingomyelin, cholesterol, lysophosphatidylcholine, arachidonic acid, fatty acids, prostaglandins and leukotrienes both in their membranes and as cargo [60]. The lipid composition of the membranes of EVs plays a crucial role in their formation and functionality. Bis(monoacylglycero)phosphate and ceramides are essential components of the lipid bilayer in exosomes and contribute to their biogenesis and structural stability [61]. In addition, the translocation of phosphatidylserine into the outer membrane layer in combination with the presence of cholesterol or sphingomyelin is necessary for the formation and release of microvesicles [62,63]. The diversity of lipid constituents within EVs is key to the specificity of their cargo-loading mechanisms. For example, the uptake of miRNA into nascent exosomes is facilitated by ceramides, while the packaging of certain proteins such as angiopoietin requires the presence of phosphatidic acid. Different lipid classes are recognized and processed by specific protein complexes, such as sphingomyelin phosphodiesterase 2/ceramide [64] and syntetin for phosphatidic acid [65]. Additionally, the presence of phosphatidylserine on exosomes and microvesicles’ outer membranes aids in their recognition by recipient cells through receptors like T cell/transmembrane, immunoglobulin and mucin (Tim1 or Tim4) receptors, promoting cellular uptake [66].

It has also been found that the lipid compositions of EVs membranes of different cell types are unique. However, the exact mechanisms through which the lipid composition of an EVs membrane influences its function are still under investigation [67]. It is important to note that the difference in the lipid composition of EVs membranes between normal and pathological cells is an important diagnostic criterion [68]. In addition, enzymes involved in lipid metabolism and contributing to their conversion into biologically active signaling molecules have been identified in the proteome of EVs [60]. For example, cluster of differentiation 14 (CD14) dendritic cells and macrophages produce small EVs containing leukotriene C4 hydrolases and synthases that are involved in the production of proinflammatory lipid mediators [69]. In vitro studies have shown that human bone marrow-derived MSCs, when cultured with polyunsaturated fatty acids such as arachidonic acid, eicosapentaenoic acid and docosahexaenoic acid, produce EVs that contain these fatty acids in their membranes. These fatty acids are known to be the precursors of eicosanoids and resolvins [70]. Furthermore, the incubation of mouse MSCs with excess docosahexaenoic acid in vitro resulted in the synthesis of resolvin D2 in MSC-EVs [71]. EVs have also been shown to contain unique lipid signaling molecules that are specific to certain cell types. For example, microglial cells produce EVs containing endocannabinoids such as N-arachidonylethanolamine and 2-arachidonylglycerol, which inhibit the neuronal release of glutamine neurotransmitters and γ-aminobutyric acid (GABA), thus regulating the balance of excitation and inhibition in the transmission of signals between neurons [72].

Increasing research on the molecular nature of EVs has led to a more sophisticated understanding of cell-to-cell communication and insights into the pathophysiological mechanisms of a number of diseases. This has fostered the development of novel diagnostic tools and therapeutic strategies that take advantage of the molecular cargo of EVs. Nevertheless, it must be recognized that this scientific field is still in its infancy. Extracellular vesicles are characterized by a multitude of hundreds of biologically active components, such as proteins, lipids, RNAs and metabolites, each of which can exert different effects. Indeed, the heterogeneity of these components can lead to a variety of outcomes, some of which may exhibit synergistic or antagonistic interactions, adding to the complexity of their biological functions. Consequently, the net biological impact of EVs may depend not only on their molecular nature but also on the current functional state of the recipient cells. Such recipient cells may interpret and respond to the vesicular signals in a context-dependent manner which is influenced by their physiological or pathophysiological state, their signaling history and the microenvironment in which they reside. However, it should be noted that this branch of research is still quite young, and further research is needed to fully understand the complexity of the mechanisms mediated by biologically active molecules in the cargo of EVs and their clinical application.

The preservation of cargo molecules within EVs is crucial as they are susceptible to degradation by external enzymes, pH variations and environmental factors during transit, potentially compromising their therapeutic efficacy. To address these challenges, the implementation of strategies to stabilize cargo molecules within EVs is essential. Methods such as encapsulating cargo in EVs with modified membrane compositions or incorporating protective proteins present innovative approaches to maintaining cargo integrity during intercellular communication in the human body [73]. Furthermore, the storage conditions of biological vesicles have a significant impact on the preservation of their cargo. Studies have shown that the cryopreservation of biological vesicles in phosphate-buffered saline (PBS) can result in mechanical damage, including ice crystal formation, pH changes, and localized regions of increased ionic strength. Similar mechanical damage can occur during the lyophilization process. As a result, ongoing research is focused on exploring and designing novel cryoprotectants for the storage of biological vesicles. Currently, Hanks’ Balanced Salt Solution supplemented with either the nonionic linear copolymer Poloxamer 188 or trehalose is considered a promising alternative to PBS for enhancing the stability of biological vesicle storage [74,75].

## 4. Mechanisms of EVs’ Interaction with Recipient Cell Membranes

EVs serve as important mediators of intercellular communication by transporting biologically active and informational molecules from donor cells to recipient cells. Once released, EVs can influence the microenvironment and be transported through body fluids to reach distant target cells. EVs can be internalized into target cells through different mechanisms which can be divided into specific (internalization) and non-specific (the integration of the EV lipid bilayer into the cell membrane structure by the interaction of the hydrophobic regions of the lipids). Internalization can occur by phagocytosis-mediated and/or micropinocytosis-mediated processes as well as by clathrin-, lipid raft- and caveolin-mediated endocytosis [76]. During endocytosis, EVs are taken up by the cell through an invagination of the plasma membrane, allowing biologically active EVs molecules to be released directly into the cytoplasm of the cell [53]. Once inside the cell, EVs release their contents of proteins, nucleic acids, lipids and metabolites, which can modulate cellular signal transduction, gene expression and cellular functions and influence a variety of physiological and pathological processes. In addition, proteins and/or sialic acid on the surfaces of EVs can mediate specific interactions with target cells. For example, the presence of the tetraspanin CD63 on the surfaces of EVs allows for specific interactions with neuronal and glial cells, whereas the absence of CD63 allows for interaction only with dendritic cells [21]. Similarly, the presence of sialic acid on the surfaces of EVs allows for specific binding to HeLa cells through an interaction with the CD33 receptor [77]. The major histocompatibility complex on the surfaces of dendritic cell-derived EVs allows them to activate T-helper cells [78]. EVs carrying TNF, Fas ligand (FasL) and tumor necrosis factor-related apoptosis-inducing ligand (TRAIL) on their surfaces are able to induce apoptosis in tumor cells if the receptors for these ligands are present on their surfaces [79].

A comprehensive understanding of the mechanisms underlying vesicular–cellular interactions is essential to elucidating their significance in both physiological and pathological states and to advance the development of targeted vesicular transport systems. For example, engineered extracellular vesicles carrying the rabies virus glycoprotein on their membrane have been used for the precise delivery of cargo to neuronal cells [80]. These exosomes efficiently transported bioactive components to the cortex and hippocampus and led to a reduction in amyloid plaque deposition, beta-amyloid levels and inflammation in transgenic mice (human amyloid precursor protein(APP)/presenilin 1 (PS1)) [81].

## 5. Sources of EVs and Methods for Their Isolation

**Figure 2:**
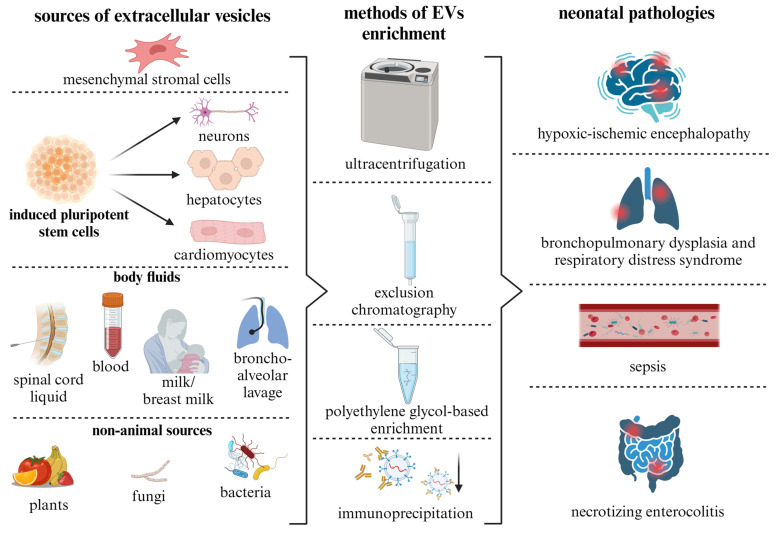
Sources of EVs and their common isolation methods for neonatal pathology therapy. Created using BioRender.com.

The therapeutic potential of EVs is mediated by their molecular cargo, which is inherited from parent cells [82]. First of all, the composition of t proteins on their surfaces determines the specificity of EVs in interacting with certain target cells. Accordingly, the proper selection of EV sources may allow for the achievement of the greatest therapeutic efficacy in relation to specific types of cells or tissues involved in the pathological process [83]. EVs can be derived from cultured, primary or genetically modified cells as well as from most biological fluids of the body (Figure 2) [84]. Additionally, intercellular communication via EVs has been demonstrated in organisms of non-animal origin, including plants, bacteria and fungi. This suggests that tissues and fluids from these species may also be potential sources of EVs [85].

The conditioned culture medium is a widely used source of EVs, which can be isolated from it using various methods [86]. The use of special culture media can increase the yield of EVs compared to standard cultivation conditions [87]. In most studies focusing on the treatment of neonatal pathologies, EVs are isolated from conditioned stem or progenitor cell culture media [88]. The most common and best-studied source of EVs are MSCs [89], which are isolated from bone marrow, adipose tissue or postpartum umbilical cords/placentas [90]. MSCs from adipose tissue represent a good source of autologous material and are suitable objects for obtaining EVs [91]. The biology of these cells is quite well studied, and therapeutic effects have been confirmed in animal models of neonatal diseases such as BPD [92], HIE [93], NEK [94], congenital retinal diseases [95] and sepsis [96]. Furthermore, EVs derived from perinatal MSC tissues have shown promising therapeutic potential for the treatment of acute pathological conditions of the brain [93,97,98]. EVs may be derived from either autologous or allogeneic sources. However, it is crucial to recognize that pathological neonatal conditions often present as acute emergencies. Experimental studies in model systems have demonstrated the therapeutic potential of EVs during these acute phases. Consequently, the use of allogeneic EVs is frequently necessitated given the time constraints associated with the cultivation of autologous MSCs and the subsequent production of vesicle preparations, which typically require 2–3 weeks.

Despite the focus on MSCs in research, alternative sources of vesicles with therapeutic effects have also been identified. Important sources of EVs may be immune cells such as T cells, B cells and macrophages. These cells release EVs that play a key role in intercellular communication, pathogenesis and immunomodulation [99]. In particular, macrophage-derived EVs with high levels of CD14 were shown to attenuate significant organ damage in a sepsis mouse model [100]. EVs derived from human endothelial progenitor cells have been associated with a reduction in organ damage in a sepsis model through the restoration of vascular permeability [101]. EVs from human and murine epicardial cells also show the ability to prevent the development of infarction when administered locally to the injury zone in newborn mice [102]. In addition, EVs derived from human plasma were found to protect neonatal rat cardiomyocytes against apoptosis under conditions of glucose and oxygen deprivation [103]. Cultures of specialized cells differentiated from induced pluripotent stem cells represent a promising EV source. For example, vesicles from neuronal and astroglial cultures have shown neuroprotective effects in experimental models [104,105,106]. In particular, EVs from intestinal neural stem cells have been associated with gastrointestinal protection against necrotizing enterocolitis in preterm rat pups [107]. Despite these findings, it remains unclear whether all human stem/progenitor or somatic cells can produce EVs with therapeutic potential. Emerging evidence suggests that the therapeutic efficacy of tissue-specific EVs may be highly dependent on the age of the donor. For example, kidney tissue-derived EVs from newborn rat pups after acute injury showed a more pronounced reparative effect on the kidneys of mice than EVs derived from adult animals [108]. A correlation was found between the increasing age of the donor and a decrease in the neuroprotective effect of bone marrow-derived MSCs [109].

Body fluids such as blood, urine, cerebrospinal fluid and amniotic fluid are valuable sources of EVs that can be collected for therapeutic purposes [90]. The use of body fluids as sources of EVs provides a non-invasive and readily available means of obtaining biologically active EVs for regenerative medicine. EVs from breast milk and amniotic fluid have shown potential for neonatal therapy [110,111,112]. However, it must be considered that despite the enriched presence of different types of EVs in biological fluids, these particles represent a mixed fraction of vesicles produced by different cell types, and the therapeutic effect depends on the donor [113], which complicates the standardization of drugs for clinical use. Also, EVs isolated from these fluids may reflect the homeostasis of the corresponding tissues and organs and potentially serve as markers for various pathological conditions [114,115,116,117].

Studies on EVs provide compelling evidence that vesicles serve as a fundamental and ancient mechanism of intercellular communication. They can facilitate the horizontal transfer of genetic information not only between different species of the animal kingdom but also between different biological kingdoms, including bacteria, fungi and plants [118,119,120]. In addition, EVs have the unique ability to cross taxonomic boundaries, providing a link between organisms from different kingdoms, such as bacteria and animals or plants and animals. This suggests that EVs represent a universal mechanism of intercellular communication for all life forms and possibly an evolutionary tool [121]. EV-mediated communication between kingdoms highlights the complex connections within biological systems and opens up possibilities for the development of new therapeutic strategies utilizing their unique biological properties. EVs derived from bacteria, fungi or plants have shown an effect on physiological processes in human cells and tissues and can also be considered potential nanocontainers for drug delivery [122].

Like animal EVs, EVs from plants, fungi and bacteria contain a wide range of biologically active compounds, anti-inflammatory molecules and nucleic acids that can have a functional effect to ensure regeneration and immunomodulation in the human body [123,124,125]; they also have low immunogenicity and high biocompatibility [126,127]. Plant-derived EVs have been shown to improve intestinal function in necrotizing colitis in mice [128]. Fungi-derived vesicles are able to interact with mammalian dendritic cells, neutrophils and macrophages and reduce the inflammatory response characteristic of neonatal pathologies [129]. In turn, bacterial vesicles, known as bacterial outer membrane vesicles, play an important role in the transport of virulence factors (toxins, enzymes and lipopolysaccharides) during infection of the host organism, promoting induction of neonatal sepsis [130].

Important factors include not only the source of EVs but also the method of their isolation from biological samples, which determines the purity of the preparation and thus the therapeutic efficacy and reproducibility of the effects. Each EVs isolation method has its own unique advantages and disadvantages. For example, differential ultracentrifugation provides high EVs yields and is relatively inexpensive but can lead to contamination with proteins and other particles [131]. In contrast, size-exclusion chromatography provides highly purified EVs populations but at the cost of a lower particle yield and longer processing time as well as possible sample dilution. Polymer-based precipitation is characterized by its efficiency and simplicity; commercially available kits further contribute to its accessibility. Nevertheless, such methods indiscriminately co-precipitate vesicles of different sizes and fail to distinguish non-vesicular entities, such as protein aggregates, from EVs [132]. Finally, immunoaffinity techniques offer unrivalled specificity through antibody-mediated selection. However, they are costly, provide lower yield of EVs and carry the risk of excluding certain vesicle subpopulations [133]. Consequently, the optimal isolation strategy depends on a variety of factors, e.g., yield, economic considerations and the duration of the process.

## 6. Experimental Studies of EVs as Therapeutic Tools for Neonatal Pathologies

### 6.1. Hypoxic–Ischemic Encephalopathy

HIE in newborns is one of the most common causes of neonatal mortality and disability. Epidemiological data suggest an incidence rate of HIE of 2 to 3 per 1000 live births, representing 6–9% of all neonatal deaths and 21–23% of neonatal deaths in full-term infants. Approximately 25% of survivors suffer from severe neurological sequelae, including cerebral palsy, seizures, intellectual disability, cognitive impairment and epilepsy [134,135]. HIE is primarily developed due to reduced cerebral blood flow and insufficient oxygen supply to the brain. The pathophysiological effects of the disease progress over time, making timely and effective treatment difficult. The pathology of HIE occurs in two phases: primary and secondary energy failure. Primary energy failure is caused by an initial decrease in cerebral blood flow leading to decreased oxygen and glucose supply, resulting in decreased mitochondrial ATP production and increased lactate production. This disrupts the function of ion channels and intracellular calcium regulatory mechanisms, leading to the excessive release of glutamate, which triggers further intracellular calcium and sodium influx and eventually leads to cell necrosis. Apoptotic cell death typically occurs a few days after the injury. In the secondary phase of energy failure, which begins 6–48 h after the initial injury, mechanisms such as oxidative stress, excitotoxicity and inflammation contribute to further damage [135]. Oxidative stress especially affects the neonatal brain due to low concentrations of antioxidants and high oxygen consumption during the transition to neonatal life [136]. Understanding the mechanisms of primary and secondary energy disturbances is crucial for effective therapeutic interventions [135]. Although therapeutic hypothermia has progressed as a neuroprotective therapy for neonates with HIE, it is associated with adverse neurodevelopmental outcomes in some infants, and its use in preterm infants is only appropriate from 35 weeks of gestation [137,138,139]. There is an urgent need to develop new therapeutic approaches to treat neonates with HIE, with cell technologies emerging as a promising direction in therapy. Such new therapeutic strategies will be crucial to improving treatment options, reducing morbidity and mortality and improving the long-term prognosis for newborns with HIE [140].

Initial studies focused on evaluating the neuroprotective potential of stem/progenitor cells for morphological and functional recovery in experimental models of HIE [141]. A number of studies have shown that the systemic administration of bone marrow-derived MSCs to neonatal rat pups with HIE improved neurological functions, increased the proliferation and differentiation of neural progenitor cells into neurons and oligodendrocytes and reduced neuroinflammation and the extent of brain tissue damage [142,143,144]. The transplantation of MSCs derived from umbilical cord blood or a human umbilical cord/placental stroma reduced the severity of brain injury and increased the survival rate of animals when modeling acute pathological conditions [145,146,147]. An analysis of the neuroprotective mechanisms of MSCs indicated a greater involvement of paracrine factors as the percentage of the engraftment of MSCs and their further differentiation in the brain were extremely low [145,148]. These observations have formed a modern paradigm for the therapeutic effect of MSCs through the secretion of EVs, which possess almost all the therapeutic properties of the cells they secrete [149,150].

Several important therapeutic effects of MSC-EVs have been identified in studies on experimental models. Many researchers have identified the immunomodulatory effects of EVs [88,151], to date the main molecular mechanism of the anti-inflammatory action of MSC-EVs is considered the modulation of the mitogen-activated protein kinase (MAPK) and nuclear factor kB (NF-kB) signaling pathways in the damaged brain. Human bone marrow-derived MSC-EVs, when administered intraperitoneally to newborn mice with induced HIE, have been shown to decrease levels of the proinflammatory cytokine TNFa and increase levels of the anti-inflammatory cytokine (TGF-β) in the brain [152]. Human umbilical cord MSC-EVs from also modulate the signaling pathway activated by Toll-like receptor 4 (TLR-4) on the surfaces of microglia by preventing the degradation of NF-kB inhibitor and inhibiting the phosphorylation of MAPK kinases. This leads to reduced microglial activity and the prevention of gliosis [93]. In particular, bone marrow-derived MSC-EVs selectively inhibit the P38MAPK/NF-kB-p65 signaling pathway in microglial cells, leading to the suppression of the transcription of inflammation-related genes such as IL-6 in brain tissue after the stereotactic injection of EVs into the lateral ventricles [153]. In an in vitro study using human umbilical cords MSC-EVs, treatment was found to increase the viability of microglial cells during glucose–oxygen deprivation modeling, reduce markers associated with pyroptosis and inhibit the release of inflammatory factors. The protective effects of MSC-EVs were associated with increased mitophagy due to the activation of Forkhead Family Transcription Factor 3a (FOXO3a) [154].

Other mechanisms of the neuroprotective effect of MSC-EVs in HIE are considered to be the restoration of the structural integrity of the blood–brain barrier (BBB), the promotion of mitophagy and an increase in the expression of neurotrophic factors. Human MSCs-EVs have been shown to carry annexin A1 on their surfaces, a molecule with anti–inflammatory activity that plays a role in maintaining the integrity of the BBB [155,156]. In a study in a mouse model of HIE, the intraperitoneal injection of MSC-EVs increased the expression of neurotrophic factors such as brain-derived neurotrophic factor (BDNF), vascular endothelial growth factor (VEGF) and epidermal growth factor (EGF) in brain tissue, which was associated with an increase in the density of neurons and blood vessels and prevented secondary brain damage [152]. Similar results were obtained in the work of Turovsky E. and co-authors in which the intranasal administration of MSC-EVs from the postpartum placenta prevented the progression of brain damage caused by hypoxia–ischemia. An analysis of the MSC-EVs proteome and a subsequent inhibitory analysis of a neuroglia culture in a glucose–oxygen deprivation model showed that the neuroprotective effect of MSC-EVs is realized via the PI3K/Akt pathway [30].

Most research on the neuroprotective mechanisms of MSC-EVs has focused on miRNAs rather than their protein cargo. Several miRNAs have been identified that may play a role in the neuroprotective properties of MSC-EVs. For example, miRNA-146a-5p, found in human umbilical cord MSC-EVs, has been shown to inhibit the activity of pro-inflammatory microglia by modulating the IL-1R-associated kinase 1 (IRAK1)/ TNFR-associated factor (TRAF) signaling pathway [40]. Another miRNA, miRNA-181b, secreted from adipose tissue-derived MSC-EVs, restored the proliferation and migration of rat brain microvascular endothelial cells in vitro [157]. In addition, miRNA-126 from a similar source of MSC-EVs was associated with the induction of neurogenesis and a reduction in apoptosis in the brain tissue of rats with induced HIE [158].

In the developing brain, EVs from resident cells play a special role. They facilitate intercellular communication between developing cell assemblies and support neuron development and neuronal network formation, synaptic plasticity, the regulation of myelination of nerve fibers and the activation of microglia [159]. Exosomes derived from M2 microglia were investigated for their potential neuroprotective effects after modeling ischemic brain injury. It was shown that neurons were able to take up these exosomes, leading to a reduction in apoptotic cell death, a decrease in infarct volume and the recovery of neurological functions. This neuroprotective effect was associated with exosomal miRNA-124, which targets the ubiquitin-specific protease 14 (USP14) gene [160]. EVs from neural progenitor cells provide neuroprotection through the transfer of miRNA-150-3p, which targets the mRNA caspase-2 to control apoptotic cell death [161].

Thus, the studies conducted show that EVs from various sources have neuroprotective properties and exert pleiotropic effects on the tissues of the developing brain, including influencing neuronal plasticity. It is worth noting that the potential therapeutic efficacy of EVs is not limited to the acute phase of brain injury but rather largely prevents the development of remote neurodegenerative consequences.

### 6.2. Respiratory Distress Syndrome and Bronchopulmonary Dysplasia

RDS and BPD are common respiratory diseases in newborns that are characterized by different pathogeneses but often occur in the same patient. RDS primarily affects premature infants due to the insufficient production of surfactant, a substance that is essential for maintaining the expansion state of the lungs. The lack of surfactant leads to alveolar collapse and impaired oxygen exchange, which in turn leads to respiratory distress. The risk of developing RDS is proportional to the duration of pregnancy so that in extremely low-birth-weight neonates born at 24 weeks, RDS occurs in 98% of cases [162]. On the other hand, BPD is a chronic lung disease that often develops in infants who require prolonged mechanical ventilation and supplemental oxygen to treat RDS. The pathogenesis of BPD includes lung injury caused by factors such as inflammation, oxidative stress and mechanical trauma during mechanical ventilation, including high-frequency ventilation. As a result, normal lung growth and development are disrupted [163]. The combination of RDS and BPD is a complex interplay of surfactant deficiency, lung tissue immaturity, inflammation and damage that contributes to respiratory distress and long-term lung complications in newborns. Each year, BPD develops in approximately 25% of infants weighing less than 1500 g worldwide, and about 70% of them suffer from a moderate or severe form, with an unfavorable outcome in 19% of cases [164,165]. One of the main methods of treating BPD are various methods of respiratory support, which only prosthetically support the function of the developing lungs but do not contribute to the regeneration and maturation of lung tissue in neonates and premature infants. Studies in animal models of BPD have shown that MSCs and their secretome contribute to the restoration of the function and structure of the developing lungs [166,167].

A detrimental factor in the ventilation therapy of newborns and premature infants is the use of gas mixtures with high oxygen contents, which inevitably leads to the development of oxidative stress in lung tissue [168]. BPD therapy with MSC-EVs has been shown to reduce the effects of oxidative stress and its markers, indicating the presence of antioxidant mechanisms [169,170]. Tissue homeostasis in the lung is also associated with the development of an intense inflammatory response leading to fibrosis, vascular remodeling and pulmonary hypertension [171,172]. The first study on the protective paracrine properties of MSCs in the development of acute neonatal pulmonary hypertension showed that the fractionation of the conditioned culture medium on exosomes contributed to a reduction in lung infiltration by macrophages and inflammation, as well as a decrease in the development of pulmonary hypertension by modulating the signal transducer and activator of transcription 3 (STAT3) signaling pathway [173]. Willis G.R. et al. demonstrated a therapeutic effect of exosomes isolated from a conditioning medium of human MSCs from an umbilical cord or bone marrow in a mouse model of BPD. The intravenous administration of these exosomes significantly attenuated the progression of hyperoxia-induced pulmonary inflammation. This intervention subsequently led to improvement in characteristic BPD manifestations, including fibrosis, vascular remodeling and pulmonary hypertension [167]. An important finding was the ability of MSC exosomes to modulate the phenotypic balance of macrophages in the lung by attenuating the pro-inflammatory M1 phenotype and enhancing an anti-inflammatory M2-like state [167]. Furthermore, MSC-EVs from the human umbilical cord have been shown to restore the population of T-regulatory lymphocytes (Treg cells) when modeling hyperoxia in newborn mice [174]. The therapeutic properties of exosomes from human umbilical cord MSCs in a hyperoxia mouse model were elucidated, with efficacy correlating with the presence of the protein TSG-6 in the exosomes. In particular, hyperoxia-induced dysfunctions in both the brain and the heart were ameliorated after the systemic administration of exosomes [175]. VEGF contained in exosomes is a potential candidate involved in lung regeneration mechanisms in BPD modeling, as exosomes without VEGF had no therapeutic effect [176,177]. The activation of hypoxia-induced factor 1-alpha (HIF-1α) could also be one of the mechanisms for the realization of a therapeutic effect through the action of EVs [169].

Alveolar epithelial cells type II (AEC-IIs) play a crucial role in the development of BPD as their apoptotic death leads to a disruption in the structural integrity of the alveoli [178]. Both in vitro and in vivo studies have shown that MSC-EVs can prevent the apoptotic death of AEC-IIs [179,180]. When MSC-EVs were administered systemically to newborn mice with induced BPD, there was a reduction in apoptotic cell death in lung tissue, as evidenced by reduced mRNA levels of the pro-apoptotic factors caspase 1 (Casp1) and the apoptosis regulator Bax [92]. Hydrogen peroxide-induced apoptotic death of the alveolar epithelial cell line RLE-6TN was prevented by treatment with bone marrow-derived MSC-EVs via mechanisms associated with miRNA-425, which targets phosphatase and tensin homolog gene (*PTEN*) and enhances the PI3K/AKT axis [181]. EVs derived from human amniotic epithelial cells (hAECs) can be used in regenerative medicine, particularly for the treatment of BPD. The influence of the gestational age of hAEC donors on the therapeutic efficacy of EVs in an experimental model of BPD was investigated. EVs from full-term infants reduced lung injury, increased the amount of AEC-IIs in the alveoli and reduced airway hyperresponsiveness, pulmonary hypertension and right ventricular hypertrophy, while hAEC-EVs from preterm infants showed no therapeutic effect [182]. The intraperitoneal administration of MSC-EVs effectively suppressed the development of pulmonary fibrosis in a model of neonatal rats with pulmonary hypertension. According to the authors, this occurred due to the EVs’ potential to inhibit the transdifferentiation of AEC-IIs into AEC-Is by downregulating the expression of WNT5a mRNA, which is a critical factor in transdifferentiation and the initiation of fibrosis [183].

### 6.3. Neonatal Sepsis

Neonatal sepsis is a systemic infection that occurs in infants up to 28 days of age and is caused by bacteria, viruses or fungi. The sources of infection may include pathogens from the mother’s genital tract with which the newborn comes into contact during birth, such as Group B *Streptococcus*, *Escherichia coli*, *Listeria monocytogenes*, *Haemophilus influenzae* and, less commonly, coagulase-negative *Staphylococcus* strains and *Streptococcus pneumoniae*. On the other hand, the infection can also be acquired in health care settings, including nosocomial antibiotic-resistant strains. The pathogenesis of sepsis involves the penetration of germs into the bloodstream, leading to bacteremia, an increased inflammatory response and even septic shock. Newborns, especially preterm infants, are highly susceptible to infections due to their immature immune system, their limited ability to localize infections and their reduced number of pre-formed antibodies. Consequently, the uncontrolled development of the immune response can lead to multiple organ failure and neonatal death, especially in the case of infection with antibiotic-resistant microorganisms [184,185]. To date, antibiotic therapy has not been able to completely combat the development of infections in newborns. An analysis of the effectiveness of antibiotic therapy has shown that the selection of an appropriate antibiotic is difficult for about 35% of neonates and preterm infants, with therapeutic measures often proving ineffective or worsening the clinical manifestations [186]. Due to their immunoregulatory and systemic regenerative effects, MSCs are promising therapeutic agents in the treatment of sepsis and associated complications in combination with basic therapy [187]. However, we could not find any relevant studies on the use of MSC-EVs for sepsis therapy in the field of neonatology. EVs have been identified as key players in the pathophysiology of sepsis, but they also show anti-inflammatory, anti-apoptotic and immunomodulatory properties in sepsis models [188]. Sepsis can lead to bacterial meningitis if the bacteria cross the BBB, which is normally impermeable to pathogens, and is life-threatening in neonates and preterm infants [189]. Kim Y. et al. demonstrated that the administration of MSCs significantly reduced bacterial growth and brain damage in neonatal rats with *E. coli*-induced meningitis. Although bacterial growth in the cerebrospinal fluid could not be significantly reduced, MSC-EVs dramatically reduced meningitis-induced brain cell death, reactive gliosis and inflammation, leading to notable decreases in the levels of pro-inflammatory cytokines IL-1β and TNF-α in the brain tissue of the experimental animals. In addition, this study found that while antibiotics alone effectively eliminated bacteria, they did not alleviate the inflammation associated with bacterial meningitis. However, when the antibiotics were combined with MSC-EVs, the inflammation and subsequent brain damage were significantly reduced. This suggests that therapy based on MSC-EVs may offer a novel approach to the treatment of neonatal meningitis by utilizing the anti-inflammatory and immunomodulatory properties of MSC-EVs [190].

### 6.4. Necrotizing Enterocolitis

NEC is a severe disease in the neonatal period characterized by inflammation and subsequent necrosis of the intestinal wall. It primarily affects premature infants and is caused by a variety of factors, including prematurity, artificial feeding, intestinal ischemia and aberrant bacterial colonization [191] Its pathogenesis involves a disruption in tissue homeostasis in the immature intestinal tissue between immune protection and the intestinal microbiota, resulting in a strong inflammatory response that damages the intestinal tissue. This damage can progress to necrosis and perforation of the bowel, requiring surgery to remove the affected bowel segment and possibly resulting in “short bowel syndrome” [192]. The primary intensive therapy includes the discontinuation of enteral nutrition (“bowel rest”), the conversion of the child to complete parenteral nutrition and the initiation of antibiotic therapy. In cases in which necrosis and perforation of the intestinal wall occur, surgical intervention may be required [193].

The effective treatment of NEC requires a comprehensive interdisciplinary approach along with personalized care. In addition, there is an ongoing need to research and develop new therapeutic strategies that directly target the primary pathogenic mechanisms of the disease. Various treatment strategies have been proposed for NEC, but recently, MSC therapy has become the preferred option due to its pronounced therapeutic efficacy. There is evidence that therapeutic effects can be achieved not only via the transplantation of stem/progenitor cells but also through the use of a conditioned culture medium containing cell secretomes and EVs [194,195,196]. In an experimental model of NEC, it was shown that bone marrow-derived MSCs and their exosomes contributed to a significant reduction in the development of the pathological process and maintained the integrity of the intestinal mucosa and its physiological permeability. Notably, within a model that simulated mechanical injury in an intestinal epithelial cell line (IEC-6), the therapeutic effects were exclusively attributed to exosomes containing in conditioned culture medium as opposed to an exosome-free conditioned culture medium [197]. McCulloh, C.J. and colleagues compared the therapeutic efficacy of EVs derived from four different types of rat stem cells in an NEC model, namely MSCs from amniotic fluid, bone marrow MSCs, neural stem cells differentiated from amniotic fluid stem cells and neonatal intestinal epithelial cells. Their results showed that EVs secreted by neural stem cells were the most effective for NEC treatment at the lowest dose studied (8 × 10^7^ EVs). Increasing the dose to 4 × 10^8^ EVs demonstrated equivalent efficacy for all four sources of EVs, resulting in a reduction in the incidence of NEC and the severity of necrotic tissue damage [107].

It is postulated that the primary pathogenetic factor in the development of NEC is an inflammatory response triggered by the immaturity of the intestinal tissue [198]. In this context, the immunoregulatory functions of MSC-EVs have been studied in detail in different models of acute intestinal injury. The systemic administration of human bone marrow-derived MSC-EVs significantly improved colitis symptoms by attenuating inflammation, maintaining intestinal barrier integrity and inducing the polarization of macrophages to the M2 phenotype while preventing intestinal fibrosis. The therapeutic effects of MSC-EVs were mediated by their interaction with colonic macrophages and the stimulation of IL-10 production by macrophages. Notably, the systemic removal of resident intestinal macrophages using a Clodronate-Liposome (Clod–Lipo) agent resulted in loss of the therapeutic effect of MSC-EVs in experimental animals. Research suggests that MSC-EVs have the potential to modulate the balance of T-cell populations toward an anti-inflammatory state. For example, the intraperitoneal administration of adipose-derived MSC-EVs in mice resulted in a restoration of Treg cell populations in the spleen and mesenteric lymph nodes, which correlated with a reduction in intestinal tissue damage in an experimental colitis model [199]. Furthermore, the intraperitoneal administration of MSC-EVs from a human umbilical cord increased the activated Th2 population and reduced the inflammatory Th1 and Th17 populations in the mesenteric lymph nodes [200] and restored the balance between Tregs and Th17 in the blood of experimental animals with colitis [201]. A direct correlation was found between the cargo of MSC-EVs and their therapeutic properties. For example, miRNA-378a-5p, carried by MSC-EVs, was found to decrease levels of NLRP3 inflammasome complex proteins in macrophages, highlighting a possible mechanism for their anti-inflammatory effects [202]. In addition, the presence of TGF-β1 in the cargo of EVs has been shown to decrease the activity and quantity of neutrophils, dendritic cells and CD4 and CD8 T lymphocytes and induce a regulatory phenotype in T cells and an immunosuppressive phenotype in dendritic cells in intestinal tissues [203]. MSC-EVs derived from perinatal and adipose tissues protect epithelial cells from apoptosis induced by the oral administration of dextran sodium sulfate (DDS). These protective effects include a reduction in ROS levels in intestinal tissue, the inhibition of myeloperoxidase activity in infiltrating neutrophils and the suppression of the mRNA synthesis of proapoptotic factors such as caspases-3, -8 and -9 [200,204]. MSC-EVs can stimulate the proliferation of intestinal epithelial cells [200,204,205]. Maintaining the integrity of the intestinal epithelial layer and preserving the tight junctions between epithelial cells are essential for the formation of the protective intestinal barrier. MSC-EVs from various sources, mainly from milk, contribute to the maintenance of this barrier by preventing the death of various types of intestinal epithelial cells, including enterocytes [204,206], intestinal stem cells [207] and goblet cells [200]. In addition, MSC-EVs can also maintain the structural integrity of the tight junctions between intestinal epithelial cells [200,205,208].

In the neonatal period, the integrity of the epithelial barrier, the immune system and the formation of the commensal microbiota in the infant’s gastrointestinal tract jointly determine the homeostasis of the intestinal mucosa. Breast milk plays a fundamental role in the regulation of the above processes, as it has been found to act in many ways through the action of breast milk-derived extracellular vesicles (MEVs) [209,210,211]. MEVs are secreted during lactation by the epithelial cells of the mammary gland [212] and possibly also by the MSCs resident in the mammary gland and constitute a significant proportion of the total fraction of milk EVs [213,214]. MEVs play an important role in the development of the neonatal immune system [215] and in the maturation of cells of the gastrointestinal tract [216]. Several studies have investigated the intravesicular contents of breast MEVs and discovered miRNAs, including let-7a, let-7b, let-7f and miRNA-148a [217], which inhibit the expression of genes that activate the NF-κB signaling pathway in dendritic cells [217,218].

A proteomic analysis of MEVs revealed the presence of proteins involved in the development of intestinal and brain tissues. Notably, human MEVs from preterm pregnancies contain elevated levels of lactadherin, which facilitates the proliferation and migration of neural stem cells and aids in the repair of damaged intestinal mucosa [219,220,221]. Additionally, high levels of peptides derived from β- and α-S1-casein were detected in the MEVs from preterm birth [219]. β-casein peptides stimulate the proliferation of CD19+ B-cells, and α-S1-casein derivatives provide protection against ROS and suppress inflammation in the gut [222,223]. In addition, lactoferrin found in both types of MEVs (from full-term and preterm pregnancies) is involved in regulating the activity of the immune system and provides antimicrobial protection [224]. Recent studies have also demonstrated the positive role of lactoferrin in regulating the proliferation of various types of intestinal cells [225].

Recent studies have shown that MEVs from different animal species can have a comparable therapeutic effect against neonatal intestinal diseases [226]. Using a DSS-induced colitis model, the efficacy of MEVs from human and bovine milk in the treatment of colitis was demonstrated. The oral administration of these MEVs resulted in less damage to the epithelial barrier, the inhibition of fibrosis and the activation of infiltrated immune cells. Furthermore, a decrease in the mRNA expression of the pro-inflammatory cytokines IL-1β, IL-6, IL-17A and IL-33 and the restoration of normal levels of commensal microorganisms were observed [227,228,229]. The therapeutic effects of EVs from cow’s milk can be attributed to the presence of a variety of factors, including conserved miRNAs, such as those of the let-7 family, which are recognized as important regulators of the immune response in mammals [217,230]. In addition, EVs from cow’s milk have been found to increase mucin 2 production and the expression of the goblet cell markers trefoil factor 3 (TFF3) in human LS174T intestinal cells [231]. The above studies underline the therapeutic and preventive potential of the use of extracellular vesicles from breast milk, especially for premature infants, and highlight the need to integrate these vesicles into infant formula.

Maintaining a normal balance of the gut microbiome in newborns is critical for the health of the gastrointestinal tract, and the disruption of this homeostasis can lead to the development of NEC [232]. Recent studies have shown the gut microbiome to autonomously regulate its microbial composition by means of interactions between intestinal bacteria and cells in the human gastrointestinal tract mediated through the outer membrane vesicles of bacteria (OMVs), as evidenced in cases of NEC [233,234]. For example, the oral administration of OMVs produced by *Akkermansia muciniphila* resulted in reduced neutrophil infiltration and the restoration of intestinal barrier integrity in mice with colitis [235,236]. In a similar model, the oral administration of OMVs from *Lactiplantibacillus plantarum* resulted in reduced serum levels of the proinflammatory cytokines IL-6, IL-1β and TNF-α and restored the balance between proinflammatory proteobacteria and anti-inflammatory bacteria such as *Escherichia* and *Muribaculaceae* [237]. In a mouse model of DSS-induced colitis, the oral administration of bacterial OMVs from *Clostridium butyricum* led to an alleviation of bacterial dysbiosis in colitis mice and to a significant reduction in the abundance of the bacterial pathogens *Escherichia coli* and *Shigella flexneri*. Coculturing these OMVs with macrophages activated by LPS resulted in the transfer of miRNA-199a-3p to them, inhibiting pro-inflammatory signals via the MAPK and NF-κB signaling pathways [238].

## 7. Discussion and Challenges of Translating EVs into Clinical Practice

The results from experimental models of neonatal and premature infant diseases discussed in this study provide promising data on the therapeutic efficacy of EVs and form a solid basis for the further development of therapeutic research and its translation into clinical practice. However, it must be recognized that several tasks need to be addressed before EVs can be widely used clinically. Firstly, the selection of an appropriate source from which to obtain EVs with high therapeutic efficacy is a key aspect influencing the efficacy of the therapy. While previous research primarily focused on MSCs as a primary tool in regenerative medicine and as a source of EVs, [239] more recent studies have broadened the spectrum of potential EV sources. Encouraging prospects are offered by milk [227] and non-animal-derived EVs [120,121].

**Figure 3:**
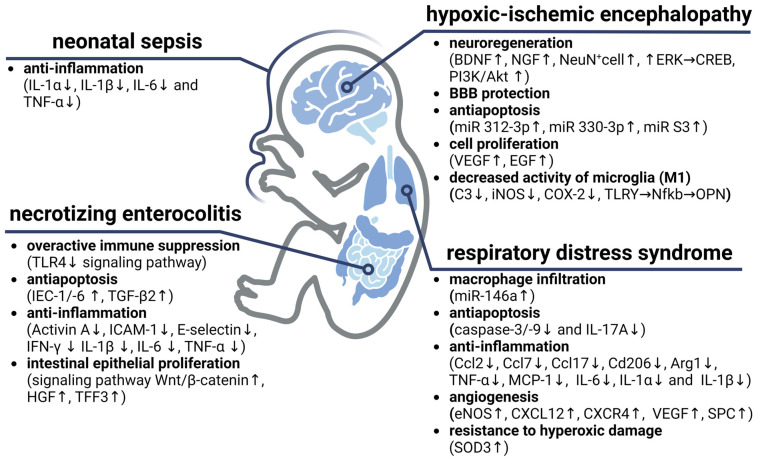
Therapeutic potential and mechanisms of EVs in neonatal diseases. ↑—upregulation, ↓—downregulation; Created using BioRender.com.

One of the biggest challenges lies in the incomplete understanding of the true nature of EVs. Although the existence of EVs and their potential therapeutic applications are known and some molecular mechanisms underlying their therapeutic effects have been explored (Figure 3), the exact identity and composition of the therapeutic components in these EVs are still not fully understood. The role of specific non-coding RNAs (such as piRNA, ribosomal RNA, small nuclear and nucleolar RNAs and circular RNAs) is not fully elucidated, and the functions of double-stranded DNA in EVs remain to be determined [239,240,241]. This knowledge gap hinders the full exploitation of the therapeutic potential of EVs and the optimization of their clinical efficacy. Further exploration of the complex composition of EVs is critical to effectively addressing these challenges. The use of advanced analytical methods, such as proteomic and genomic profiling, will facilitate the elucidation of the specific “cargo” contained in EVs. Revealing the identity and abundance of these therapeutically active components will provide a deeper understanding of how EVs exerts their therapeutic effects at the molecular level.

The standardization of protocols for the isolation, purification and characterization of EVs is essential to ensure the reproducibility and comparability of results across studies. This standardization will facilitate the development of reliable guidelines for the clinical use of EVs. The problem of the heterogeneity of EVs raises concerns about their efficacy for clinical use. To address this issue, it is crucial to develop and implement a standardized approach for the isolation of EVs [242]. In addition, it is important to establish standards for the evaluation of the qualitative and quantitative characteristics of EVs and their contents [243]. The presence of therapeutically useful cargo in extracellular vesicles implies the question of their dosage, which has a direct impact not only on the efficacy but also on the safety of EVs use. Various methods are used to quantify EVs, including assessments of the number of parent cells (cell equivalents) and the total protein concentration in EVs and specific analytical methods (the detection of particle concentration and size) [244]. However, the existing in vivo studies, including in neonatology, were performed without a standardized dosage, frequency and method of administration of EVs, which makes it difficult to determine a safe and effective dosage for clinical trials for the treatment of neonatal pathologies. Despite extensive discussions on the determination of the optimal therapeutic dose, a standardized approach has not yet been developed, partly because there are not enough experimental data. It is noteworthy that during a discussion involving a group of researchers, the term “therapeutic unit” was proposed as a standard for calculating the dose, which we discussed in detail in the “Methods” section. We have used this approach to standardize the therapeutic doses used in the reviewed papers, which are listed in Table 1. This approach allows the therapeutic doses to be compared with each other. As can be seen, the doses in the different studies can vary by up to 100-fold.

It is also important to investigate all aspects of the potential side effects of EVs. Research has shown that exosomes can carry tissue factor and phosphatidylserine, which activate blood coagulation processes, on their surfaces [260,261]. Moreover, the acute, rare thrombotic reactions observed in patients after MSC administration in clinical trials are attributed to side effects associated with vascular thrombosis and the subsequent inflammatory response [262]. It is also a crucial factor to consider during the development of sepsis-related disseminated intravascular coagulation (DIC), in which tissue factor on the surfaces of platelets plays a leading role [263]. Tissue factor is recognized as a critical regulator of hemostasis capable of initiating localized thrombosis under specific circumstances, thereby eliciting an inflammatory immune response [264]. Despite ongoing efforts to elucidate the mechanisms underlying sepsis-induced DIC, clinicians worldwide continue to encounter challenges in effectively managing this condition. As such, there is a pressing need for innovative treatment strategies [265]. It is worth noting that EVs may have promise in this regard, but further research is warranted to evaluate the safety of EVs in relation to thrombotic events. Our previous work showed that pre-incubating EVs with heparin can mitigate their prothrombotic properties [260].Consequently, there is a pressing need for researchers and clinicians to develop standardized and clinically applicable protocols that are focused on the delivery methods and preparation of EV-based therapeutics and are supported by solid preclinical evidence. [266] In addition, the outcomes of combinatorial therapies require more thorough analyses. For example, an in vivo study using the HIE model in neonatal mice has shown that pretreatment with hypothermia can reduce the efficacy of subsequent cell therapy [267].

Despite all these unresolved issues, according to the clinicaltrials.gov database, three clinical trials with EVs in neonatology have been registered and are active. Study NCT03857841 is dedicated to investigating the therapeutic efficacy and safety of allogeneic bone marrow-derived MSCs-EVs in neonates aged 3–14 days with a diagnosis of BPD. A study from Russia is investigating the use of MSC-EVs to treat children with extremely low body weight in order to prevent the development of brain damage associated with prematurity (NCT05490173). The third clinical trial will evaluate the efficacy of allogeneic MSC-EVs in neonates with HIE (NCT02854579). The introduction of a broad spectrum of EV-based treatment methods into clinical practice can therefore potentially not only reduce the mortality rate in premature newborns but also significantly reduce disabilities.

## 8. Conclusions

EVs have shown promise as therapeutic agents for a variety of neonatal diseases. This promise is primarily attributed to the therapeutic potential of vesicles derived from MSCs. Despite significant progress in this field, our understanding of the molecular composition of EVs and the mechanisms underlying their therapeutic effects is still incomplete. To close this knowledge gap, it is essential to perform comprehensive analyses of vesicle composition using omics technologies that comply with Good Laboratory Practice (GLP) standards. In addition, it is critical to conduct rigorous preclinical safety and efficacy studies with well-characterized vesicles that comply with the MISEV guidelines. We assume that adopting a careful research design during the preclinical phase will be critical to facilitating the seamless transition of these findings into initial clinical investigations.

## Figures and Tables

**Table 1:** Overview of studies investigating the therapeutic potential of EVs in neonatal diseases.

References	Sources of EVs	Model	Protocol of Administration	Dosing	Therapeutic Effects	Elucidated Mechanisms
**HYPOXIC–ISCHEMIC ENCEPHALOPATHY**						
[245] 2016	human bone marrow MSCs	the Rice–Vannucci model	total dose—2.4 × 10^10^ EVs route of administration—intravenous administrations per day—2 days—1	1 unit *	↓ total number and duration of seizures ↓ pathological fluctuations of blood pressure	↑ baroreflex-mediated heart rate response
[156] 2019	human bone marrow MSCs	the Rice–Vannucci model	total dose—2.4 × 10^10^ EVs route of administration—intravenous administrations per day—2 days—1	1 unit	↓ permeability of blood–brain barrier	↑ Annexin A1/FPR in neonatal brain endothelial cells and microglia
[151] 2019	human bone marrow MSCs	the Rice–Vannucci model	total dose—1.25 × 10^9^ EVs route of administration—intranasal administrations per day—1 days—1	0.1 units	↓ of tissue loss ↓ % of cell death ↓ microglial activation ↑ behavioral outcomes (negative geotaxis test)	
[93] 2019	human Wharton’s jelly MSCs	the Rice–Vannucci model + intraperitoneal injection of LPS	total dose—325 µg of EV protein per animal route of administration—intranasal administrations per day—1 days—1	0.3 units	↓ microgliosis ↓ neuroinflammation	↓ LPS/TLR4 signaling in microglia
[246] 2020	rat bone marrow MSCs (H2S preconditioning)	the Rice–Vannucci model	total dose—1.5 × 10^8^ EVs route of administration—intracardial injection administrations per day—1 days—1	0.06 units	↓ water content and infarct volume of the brain ↓ % of cell apoptosis ↑polarization toward the anti-inflammatory M2 phenotype ↑ memory function	↑ miR-7b-5p ↓ FOS → ↓ Iba1+ in microglia
[152] 2020	human bone marrow MSCs	the Rice–Vannucci model	total dose—2.7 × 10^8^ EVs route of administration—intraperitoneal administrations per day—1 days—3 (1,3,5 post HI)	0.03 units	↓ striatal tissue loss ↓ M1 micro- and A1 astroglia activation ↑ neurogenensis and angiogenesis ↑ myelination	
[247] 2021	mice bone marrow MSCs	the Rice–Vannucci model	total dose—100 µg of EV protein route of administration—intracardial injection administrations per day—1 days—1	0.1 units	↓ HI-induced edema, infarction, infiltrating monocytes ↓ phagocytosis of viable neurons ↑ synaptic densities	↓ p-NF-κB → ↓OPN → ↓ Iba1 in M1 microglia
[248] 2021	mice bone marrow MSCs	the Rice–Vannucci model	total dose—5 µg of EV protein route of administration—intranasal administrations per day—1 days—1	0.005 units	↓ injury volumes ↓ microglial activation ↓ neuroinflammation	↓ Iba1 → ↓ Casp3 in microglia
[249] 2021	rat primary astrocytes (P1)	the Rice–Vannucci model	total dose—2.5 µg of EVs protein route of administration—intraperitoneal administrations per day—1 days—1	0.0024 units	↓ the area of cerebral infarction ↓ HIBD-induced neuronal apoptosis ↓ oxidative stress ↓ neuroinflammation ↑ body weight ↑ cognitive functions(grip test, negative geotaxis test	↑ miR-17-5p → ↓ BNIP → ↓ Bax in brain tissue
[250] 2022	brain tissues of neonatal mice (P9) after HI	the Rice–Vannucci model	total dose—8 × 10^9^ EVs route of administration—intranasal administrations per day—2 days—1	0.066 units	↓ infarct size ↓ Casp3 expression	↑ miR-342-3p and miR-330-3p in brain tissue
[251] 2022	mice bone marrow MSCs	the Rice–Vannucci model	total dose—2 × 10^9^ EVs route of administration—intranasal administrations per day—1 days—1	0.2 units	↑ animal survival ↓ infarct volume of brain ↓ % of apoptosis cells ↓ neuroinflammation ↑ proprioceptive function	↑ miR-93 → ↓ JMJD3 → ↑ KLF2 → ↓ Casp3,Bax in neurons
[252] 2023	immortalized human bone marrow MSCs	the Rice–Vannucci model	total dose—2.7 × 10^8^ EVs route of administration—intranasal administrations per day—1 days—3 (1,3,5 post HI)	0.03 units	↑ neurogenesis and angiogenesis ↓ monocyte infiltration ↓ astrogliosis and microgliosis	
**BRONCHOPULMONARY DYSPLASIA**						
[167] 2018	human Wharton’s jelly MSCs	hyperoxia (HYRX)-induced BPD mice model (P1–P7 75% O_2_)	total dose—0.9 µg of EV protein route of administration—intravenous administrations per day—1 days—1	0.001 units	↑ lung architecture ↓ lung fibrosis ↓ peripheral pulmonary arterial remodeling	
[175] 2018	preterm human Wharton’s jelly MSCs	HYRX-induced BPD mice model (c P1–P4 95% O_2_)	total dose—4.5 × 10^8^ EVs route of administration—intraperitoneal administrations per day—1 days—2 (P2 and P4)	0.038 units	↑ lung architecture ↓ infiltration of neutrophils ↓ pulmonary hypertension ↓ alveolar-capillary leak	↑ TSG-6 signaling in lung tissue
[253] 2018	human Wharton’s jelly MSCs	HYRX-induced BPD rat model (P1–P14 60% O_2_)	total dose—0.213 × 10^10^ EVs route of administration—intratracheal administrations per day—1 days—3 (P3, P7 and P10)	0.27 units	↑ alveolar development ↓ pulmonary vascular remodeling	
[176] 2018	rat bone marrow MSCs	HYRX-induced BPD rat model (P0–P14 85% O_2_)	total dose—3.4 × 10^9^ EVs route of administration—intraperitoneal administrations per day—1 days—14 (P1–P15)	1.96 units	↑ alveolar growth ↑ lung blood vessel density ↓ pulmonary hypertension	↑ VEGF signaling in lung tissue
[177] 2018	human umbilical cord blood MSCs	HYRX-induced BPD rat model (P1–P14 90% O_2_)	total dose—20 µg of EV protein route of administration—intratracheal administrations per day—1 Days—1 (P5)	0.019 units	↑ alveolarization and angiogenesis	↑ VEGF signaling in lung tissue
[254] 2020	human Wharton’s jelly MSCs	HYRX-induced BPD mice model (P0–P14 75% O_2_)	total dose—6 × 10^8^ EVs route of administration—intravenous administrations per day—1 days—1 (PN4)	0.025 units	↓ alveolar simplification ↓ septal collagen disposition ↑ blood vessel count ↓ pulmonary hypertension ↑ functional exercise capacity	
[255] 2021	human bone marrow MSCs.	In utero induced BPD rat model (antenatal injection of *E. coli* endotoxin e20)	total dose—0.25 × 10^6^ EVs route of administration— intra-amniotic administrations per day—10 per pregnant rat days—1 (e20)	0.17 units	↓ lung simplification ↑ vascularization ↓ pulmonary hypertension ↑ lung mechanical function	
[174] 2021	human Wharton’s jelly MSCs	HYRX-induced BPD mice model (P–P7 75% O_2_)	total dose—6 × 10^8^ EVs route of administration—intravenous administrations per day—1 days—1 (PN4)	0.025 units	↑ thymic development ↑ proportion of CD4+FoxP3+ regulatory T cells ↓ alveolar simplification	
[253] 2021	human umbilical cord blood MSCs	HYRX-induced BPD rat model (P1–P14 60% O_2_)	total dose—0.64 × 10^10^ EVs route of administration—intratracheal administrations per day—1 days—4 (P3, P7, P10 and P21)	0.27 units	↑ alveolar development ↓ deposition of fibrous tissue ↑ density of M2 macrophages ↓ pulmonary hypertension	
[111] 2021	amniotic fluid-derived EVs (full-term cesarean sections)	HYRX-induced BPD rat model (P1–P14 85% O_2_)	total dose—1 × 10^10^ EVs route of administration—intratracheal administrations per day—1 days—1 (P3)	0.42 units	↑ alveolar development ↓ pulmonary hypertension	
[256] 2022	human breast milk-derived EVs	HYRX-induced BPD rat model (P1–P7 85% O_2_)	total dose—140 µg of EV protein route of administration—intragastric administrations per day—1 days—1 (PN7)	0.133 units	↓ lung tissue collapse ↓ cleaved caspase 3	↓ IL-17/↓ FADD in Type II alveolar epitheliocytes
[171] 2022	human Wharton’s jelly MSCs	HYRX-induced BPD rat model (P1–P14 85% O_2_)	total dose—96 × 10^8^ EVs route of administration—intratracheal administrations per day—1 days—1 (PN3)	0.04 units	↑ lung vascular density and alveolar structure ↓ lung inflammation ↓ pulmonary hypertension	↑ VEGF/eNOS in lung tissue
[182] 2022	human amniotic epithelial cells (term birth after caesarean sections)	in utero induced BPD mice model (injection of LPS e16) + (P3.5–P28 65% O_2_)	total dose—10 µg of EV protein route of administration—intravenous administrations per day—1 days—1 (PN4)	0.01 units	↑ lung tissue-to-air space ratio ↓ lung inflammation ↑ type II alveolar epithelial cell ↓ pulmonary hypertension ↑ lung tissue elasticity	
[179] 2022	human Wharton’s jelly MSCs	HYRX-induced BPD mice model (injection of LPS P7/P8) + 40% O_2_ P10	total dose—1 × 10^6^ EVs route of administration—intratracheal administrations per day—1 days—1 (PN9)	0.0002 units	↑ lung architecture ↑ blood vessel density ↑ mRNA of antiinflammatory cytokines in lung tissue	
[257] 2023	human umbilical cord blood MSCs	HYRX-induced BPD mice model (P1–P14 85% O_2_)	total dose—15 × 10^5^ EVs route of administration—intraperitoneal administrations per day—1 days—3 (P4–P6)	0.000063 units	↓ lung fibrosis ↑ vascular development	↑ miR-185-5p →↓ CDK6 → ↑ angiogenesis in lung tissue
[169] 2023	human bone marrow MSCs	hypoxia—induced BPD rat model (10 min 40% O_2_ + 2 min 1% O_2_ 12 times daily P1–P14)	total dose—2 × 10^5^ EVs route of administration—intraperitoneal administrations per day—1 days—14 (P1–P14)	0.00012 units	↓ simplified alveolar structure ↓ pulmonary hypertension ↑ capillary distribution ↑ respiratory efficiency ↓ oxidative stress	↑ PI3K/AKT → ↑ SOD in lung tisue
**NECROTIZING ENTEROCOLITIS**						
[197] 2016	mice bone marrow MSCs	NEC—induced preterm mice model, Barlow et al. [258] (21e) + 90 s 100% N_2_ + 4 °C 10 min twice daily (P1–P4)	total dose—2.5 × 10^9^ EVs route of administration—intraperitoneal administrations per day—1 days—1 (prior NEC)	0.1 units	↓ the overall incidence of NEC ↑ gut barrier function	
[107] 2018	neonatal mice enteric neuronal stem cells	NEC—induced preterm rat model, Barlow et al. [258] (21e) + (90 s 100% N_2_ + 4 °C 10 min every 8 h + LPS every 4 h (P1–P4)	total dose—4 × 10^8^ EVs route of administration—intraperitoneal administrations per day—1 days—1 (prior NEC)	0.017 units	↓ intestine villus destruction ↓ the overall incidence of NEC	
[231] 2019	bovine milk-derived EVs	NEC—induced preterm mice model, Barlow et al. [258] (10 min 5% O_2_ 3 times between P5–P9 + LPS 4 times between P6 and P7)	total dose—1.2 mg of EV protein route of administration—intragastric via gavage administrations per day—3 days—5 (P5–P9)	1.14 units	↑ intestine villus destruction ↑ number of goblet cells ↓ intestinal mucosal inflammation ↓ oxidative stress	
[227] 2019	human breast milk-derived EVs	NEC—induced preterm rat model, Barlow et al. [258] (21e) 90 s 1.5% O_2_ + 4 °C 10 min 3 times daily P1–P4 + LPS 1 time P1	total dose—2.4 × 10^10^ EVs route of administration—intragastric via gavage administrations per day—6 days—4 (P1–P4)	1 unit	↓ villus destruction ↓ the overall incidence of NEC	
[194] 2020	rat amniotic fluid CD117 stem cells (e14.5)	NEC—induced preterm mice model, Barlow et al. [258] (10 min 5% O_2_ 3 times between P5–P9 + LPS 4 times between P6–P7)	total dose—3.5 × 10^8^ EVs route of administration—intraperitoneal administrations per day—1 days—2 (P6–P7)	0.015 units	↑ gut epithelial regeneration ↓ intestinal inflammation	↑ Wnt/β-catenin → increased intestinal epithelial proliferation
[259] 2022	human breast milk-derived EVs	NEC—induced preterm mice model, Barlow et al. [258] (1 min 100% N_2_ + 4 °C 5 min twice a day P6–P10)	total dose—30 µg of EV protein route of administration—intragastric via gavage administrations per day—3 days—3 (P8–P10)	0.04 units	↑ ileal crypts number ↓ inflammatory microenvironment	
**NEONATAL MENINGITIS**						
[190] 2022	human Wharton’s jelly-derived MSCs after thrombin preconditioning	*Escherichia coli*—induced meningitis in newborn rats (P11)	total dose—2.6 × 10^7^ EVs route of administration—intraventricular administrations per day—1 days—1 (P11)	0.001 units	↓ neural cell death ↓ number of active microglia ↓ levels of inflammatory cytokines in brain tissues	

* the yield of EVs derived from supernatants of 4 × 10^7^ MSCs that were cultured for 48 h was defined as 1 unit; 1 unit contains 2.4 × 10^10^ EVs or 1.05 mg of EV protein; ↓—downregulation, ↑—upregulation [14].

## Data Availability

No new data were created or analyzed in this study. Data sharing is not applicable to this article.

## Supplementary Materials

The supporting information can be downloaded at https://www.mdpi.com/article/10.3390/ijms25052879/s1.
