# Supplementary figures and images for: A Review of the Use of Extracellular Vesicles in the Treatment of Neonatal Diseases: Current State and Problems with Translation to the Clinic
Source: Int J Mol Sci. 2024 Mar 1;25(5):2879. doi: 10.3390/ijms25052879 (PMC10932115; doi:10.3390/ijms25052879)

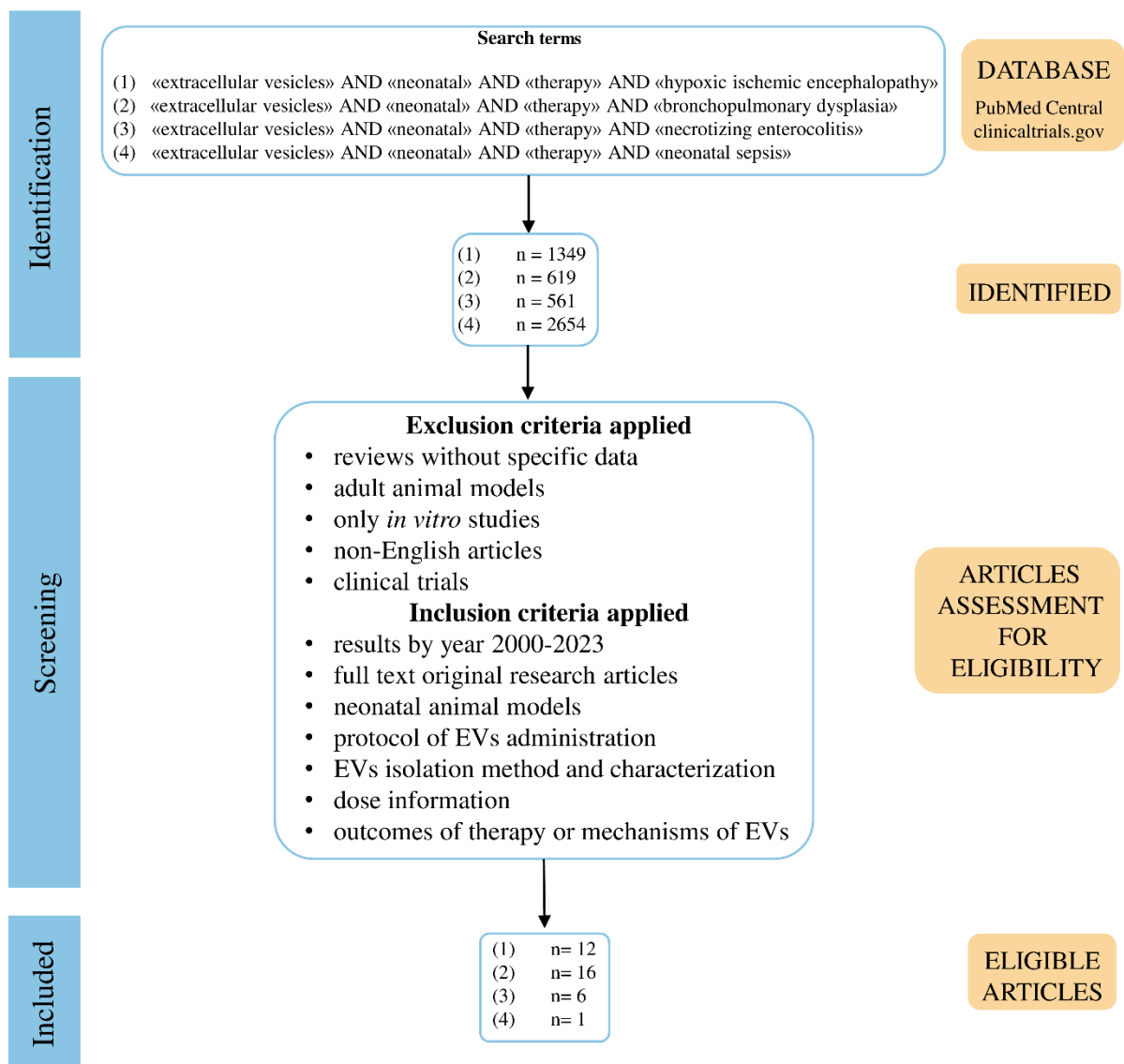

**Figure S1.** Flowchart of article selection for Table 1.

Supplement: Supplementary file 1 [file ijms-25-02879-s001.zip › ijms-2849683-supplementary.pdf]
